# Supplementary material for: Effect of current density on the solid electrolyte interphase formation at the lithium∣Li6PS5Cl interface
Source: Nat Commun. 2022 Nov 24;13:7237. doi: 10.1038/s41467-022-34855-9 (PMC9700819; doi:10.1038/s41467-022-34855-9)
Supplement: Supplementary file 1 — Supplementary Information [file 41467_2022_34855_MOESM1_ESM.pdf]

Supplementary Information:

Effect of current density on the solid electrolyte interphase formation at the lithium|Li_6PS_5Cl interface

Sudarshan Narayanan[1,2], Ulderico Ulissi[3], Joshua S. Gibson[1,2], Yvonne A. Chart[1,2], Robert S. Weatherup[1,2], and Mauro Pasta[1,2,*]

[1]Department of Materials, University of Oxford, Oxford OX1 3PH, U.K.
[2]The Faraday Institution Quad One, Harwell Science and Innovation Campus, Didcot OX11 0RA, U.K.
[3]Nissan Technical Centre Europe, Moulsoe Road, Cranfield Technology Park, Bedford MK43 0DB, U.K.
[*]mauro.pasta@materials.ox.ac.uk

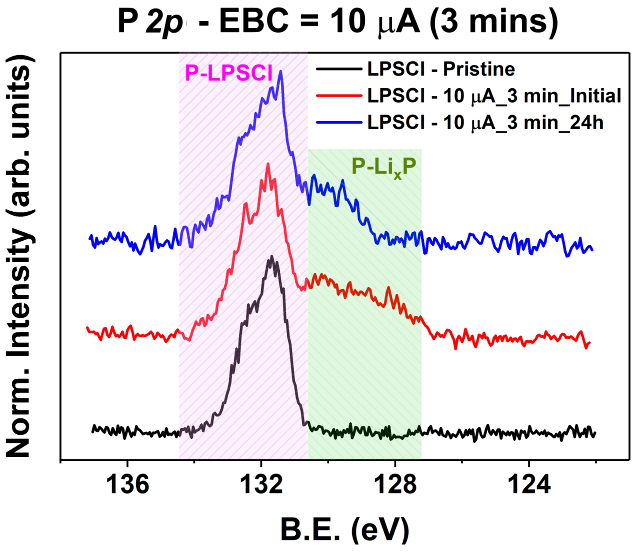

Supplementary Figure 1. Temporal stability of partially reduced Li_xP induced by virtual electrode plating process. Core-level P *2p* spectra following the virtual electrode plating process exposing the LPSCl surface with an electron beam current of 10 μA for 3 minutes. Following this exposure, a shoulder in the spectra appearing at a lower binding energy can be associated with the presence of partially reduced Li_xP species. A comparison of the spectra immediately after plating, and after a 24 hour period indicates reasonable stability of this decomposition product.

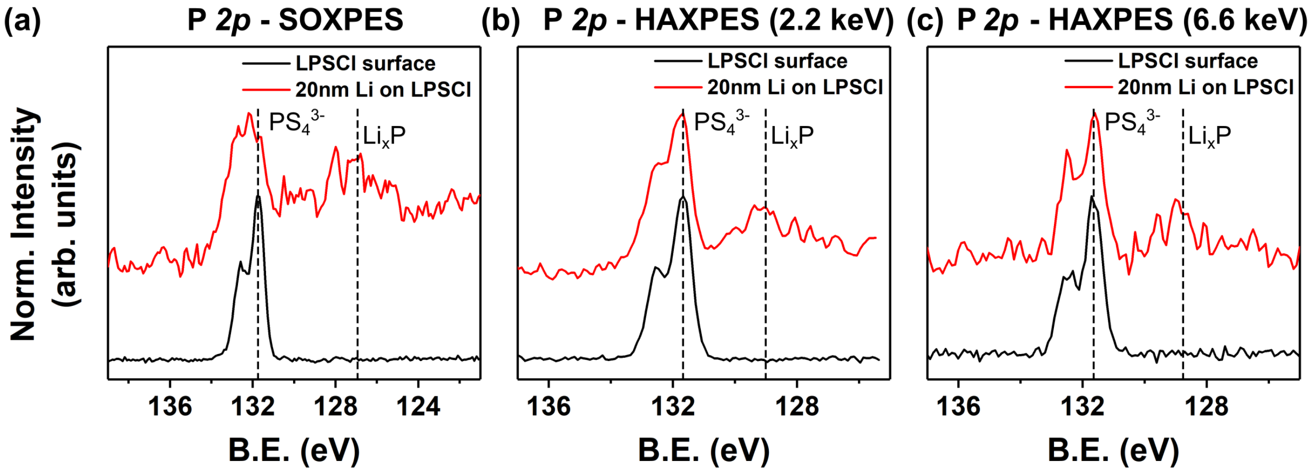

Supplementary Figure 2. Synchrotron XPS measurements. Core-level P *2p* spectra for LPSCl in the uncoated state and with 20 nm of evaporated Li film using (a) SOXPES, SOft X-ray Photo-Emission Spectroscopy, HAXPES, HArd X-ray Photo-Emission Spectroscopy at (b) 2.2 keV and at (c) 6.6 keV acquired at the I09 Synchrotron beamline in Diamond Light Source, Didcot, Oxford. The presence of a partially reduced Li_xP feature even at a depth of $\sim$15-20 nm indicates that the Li_xP species is indeed a stable decomposition product from the reaction of Li metal with LPSCl. Changes in signal-to-noise ratio occur as a result of differences in both the probing depth of the ejected photoelectron (lower energy - lower probing depth) and the absorption cross section for different photon energies (higher energy - lower cross section)

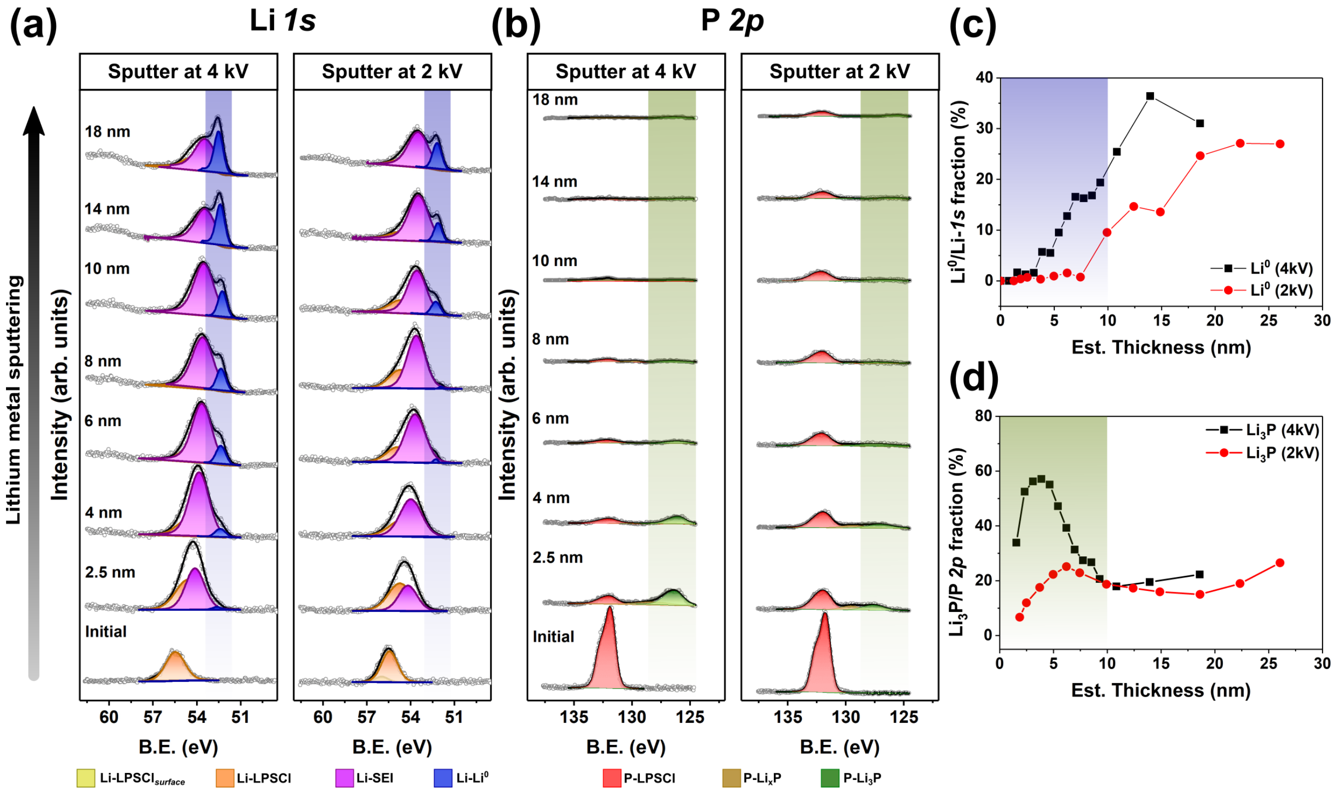

Supplementary Figure 3. **SEI evolution using in situ Li sputtering inside XPS.** Evolution of core-level XPS spectra during in situ Li sputtering at the LPSCl surface, at Ar^+ ion beam acceleration voltages of 4 kV (left panel) and 2 kV (right panel) for (a) Li *1s* and (b) P *2p* transitions, as a function of estimated deposited Li thickness. Quantification of XPS spectra plotted against estimated thickness of Li deposited, depicting compositional fractions of (c) metallic Li (Li^0) in Li *1s*, and (d) Li_3P in P *2p*. A larger fraction of Li^0 and Li_3P for same thickness of Li sputtered (highlighted by regions coloured in blue and green, respectively) reiterates the role of reaction kinetics in determining SEI composition and onset of subsequent metallisation from extended sputtering. Error bars indicate residual standard deviation between raw data and fitted curves, as computed by CasaXPS.[1]

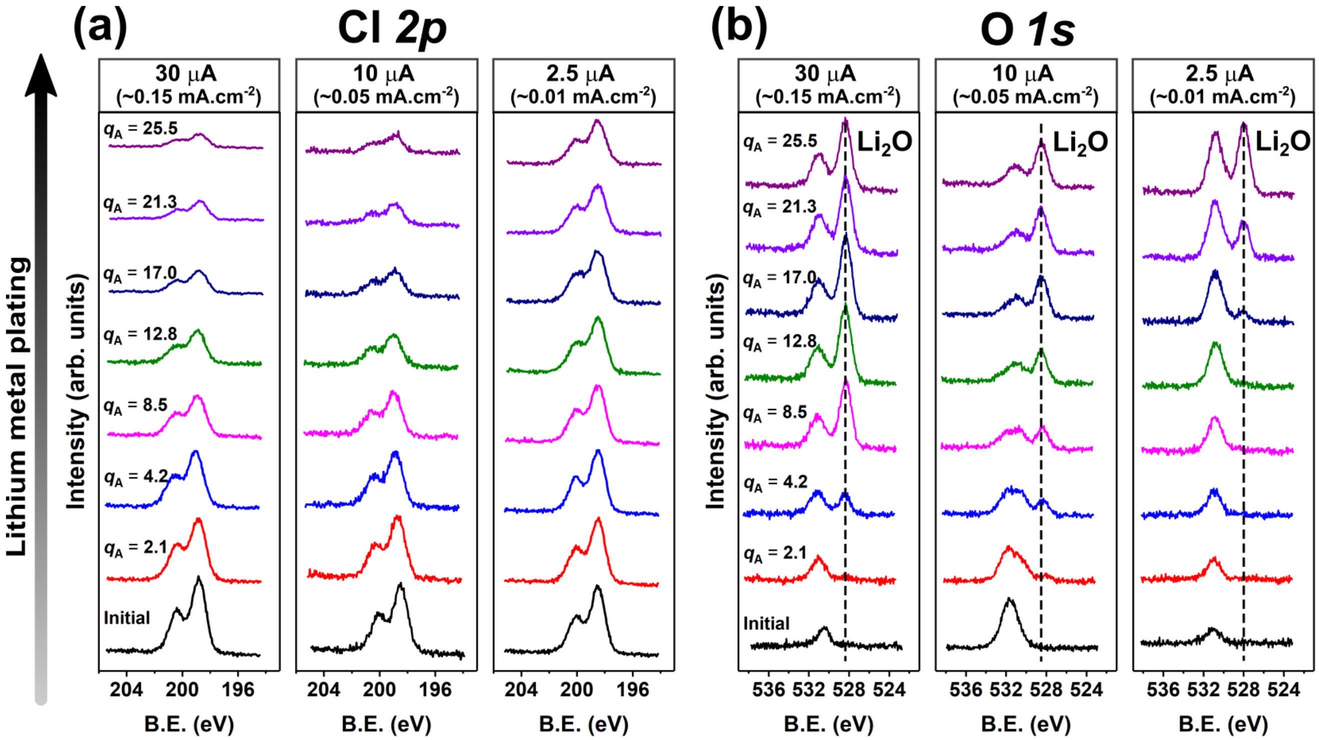

Supplementary Figure 4. Additional XPS spectra for Cl *2p* and O *1s*. Core-level XPS spectra for (a) Cl *2p* and (b) O *1s* transitions during the virtual electrode plating process conducted at electron beam currents of 30 μA (~0.15 mA·cm^{-2}, left panel), 10 μA (~0.05 mA·cm^{-2}, central panel) and 2.5 μA (~0.01 mA·cm^{-2}, right panel). No significant shifts in the Cl *2p* peaks (panel a) are observed, as has been reported previously.[2] Similarly, O *1s* spectra (panel b) demonstrate the appearance of a low B.E. feature around 528.5 eV representative of formation of Li_2O, which proceeds from Li plating. Diminishing intensity of the Cl *2p* peaks and growing Li_2O features are both indicative of the formation of a metallic Li layer. As seen here, both occur faster at higher current densities, indicating plating of metallic Li over a relatively thinner SEI layer.

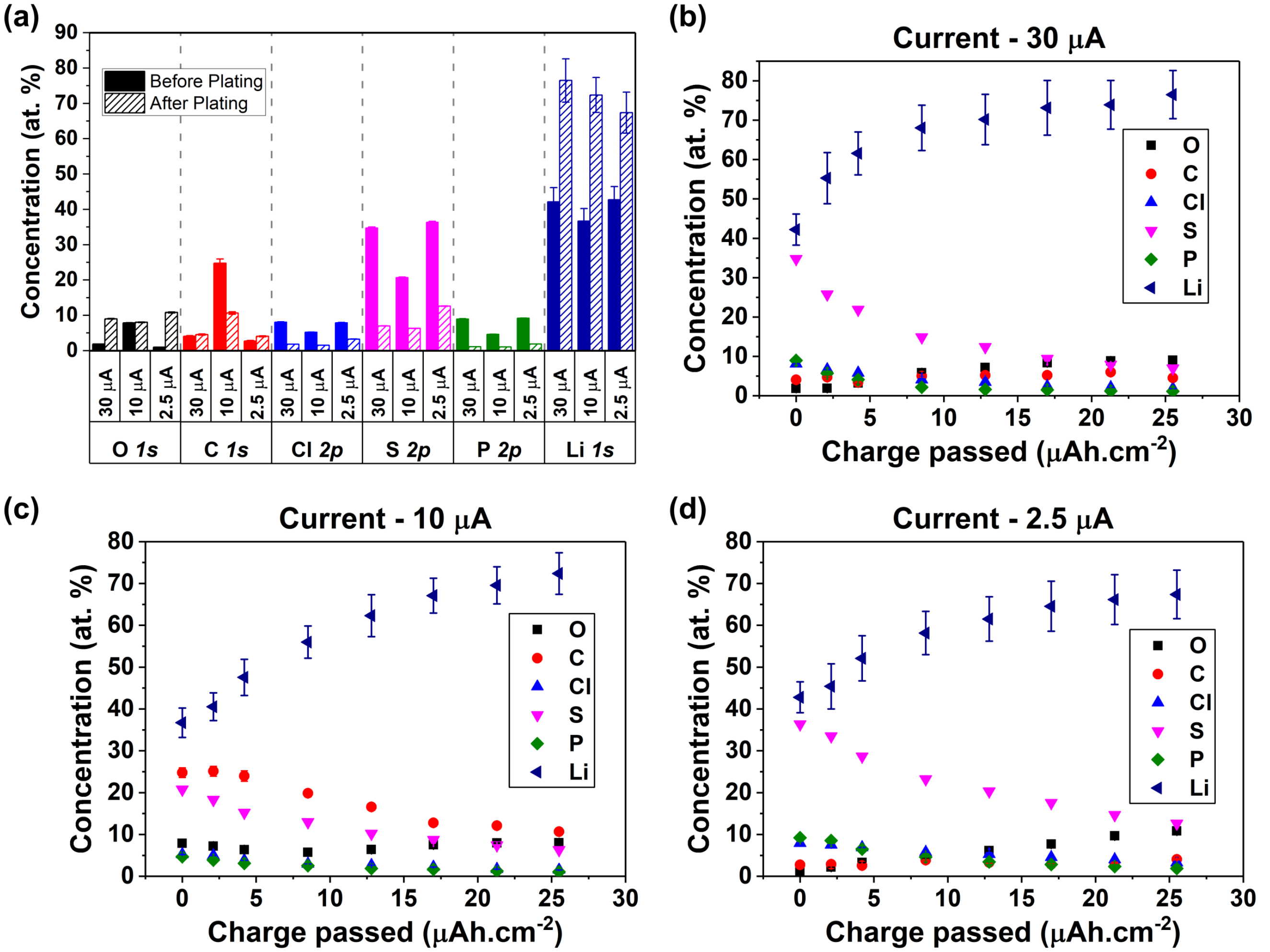

Supplementary Figure 5. Quantification of SE surface composition using XPS. (a) Comparison of the SE surface composition before and after plating of Li (∼25.5 μAh·cm^{-2}) estimated by quantifying regions from XPS spectra for O *1s*, C *1s*, Cl *2p*, S *2p*, P *2p* and Li *1s* transitions. Evolution of SE surface composition as a function of charge passed (μAh·cm^{-2}) during the virtual electrode plating process for applied EBC of (b) 30 μA, (c) 10 μA and (d) 2.5 μA. Error bars indicate errors in composition based on residual standard deviation between raw data and fitted photoemission intensities, as computed by CasaXPS.[1]

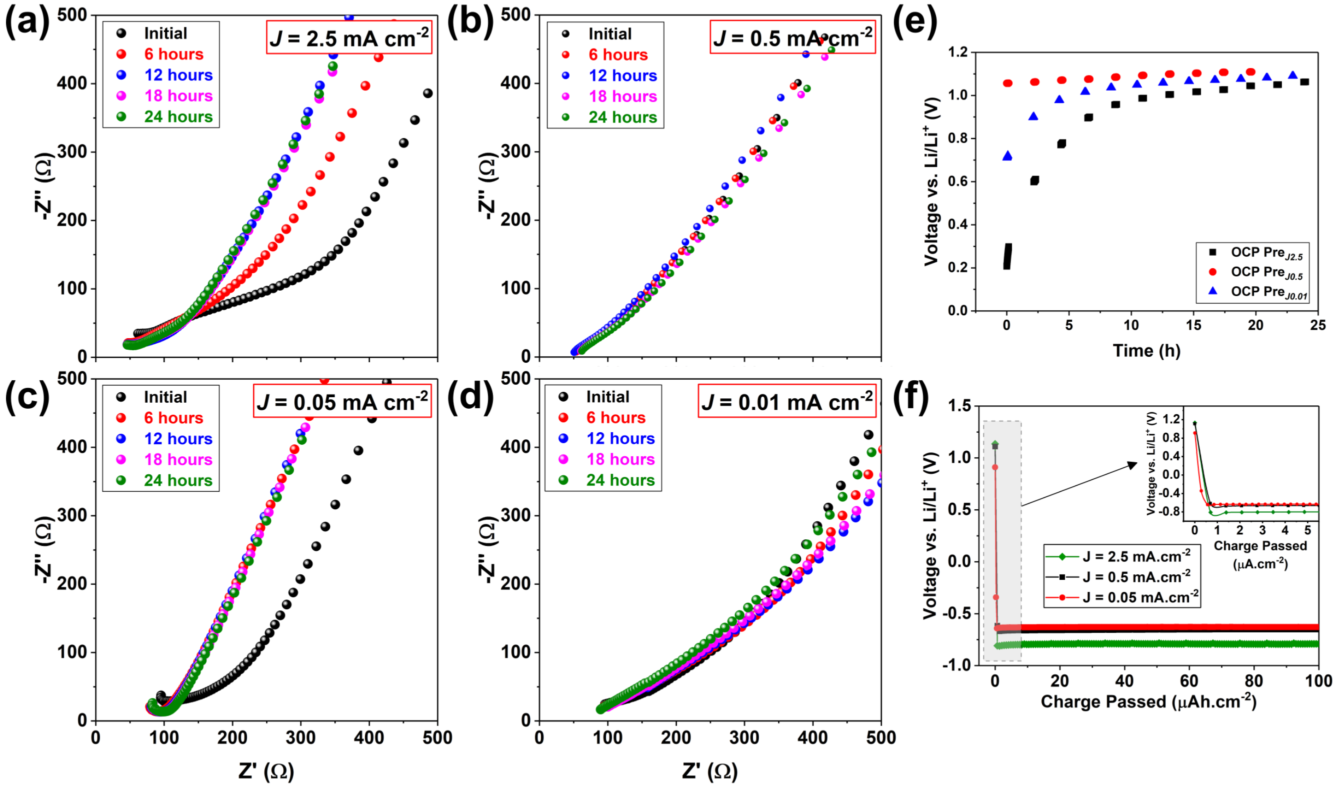

Supplementary Figure 6. Initial EIS and OCV stability measurements. EIS curves for samples used for current density variation study over a 24 hour period prior to the plating experiment being conducted, to allow for initial SEI and interfaces to stabilise, prior to plating Li at (a) 2.5, (b) 0.5, (c) 0.05, and (d) 0.01 mA·cm^{-2}. (e) Open circuit voltage (OCV) stabilities measured prior to conduct of plating experiment at various current densities. (f) Potential across the lithium-free cell SS|LPSCl|LiIn measured during plating conducted at 2.5 mA·cm^{-2}, 0.5 mA·cm^{-2} and 0.05 mA·cm^{-2}. Inset shows variations in initial overpotentials observed during the plating experiment. Voltages seen to stabilise to ∼-0.63V, the standard redox potential for LiIn vs. Li.

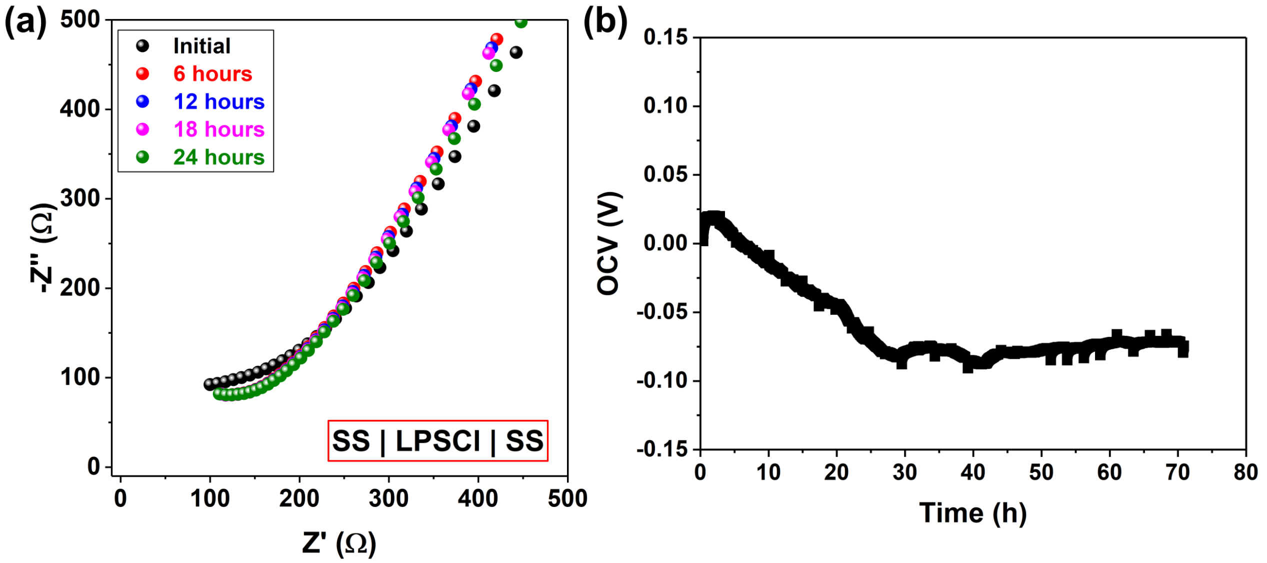

Supplementary Figure 7. **EIS and OCV stability measurements in symmetric SS|SE|SS cells.** (a) EIS spectra for symmetric SS|Li_6PS_5Cl|SS cells acquired over a 24 hour period demonstrating stabilisation of LPSCl with ion-blocking SS electrodes, at OCV conditions. (b) OCV stability measured across the same symmetric SS|LPSCl|SS cell over a 72 hour period.

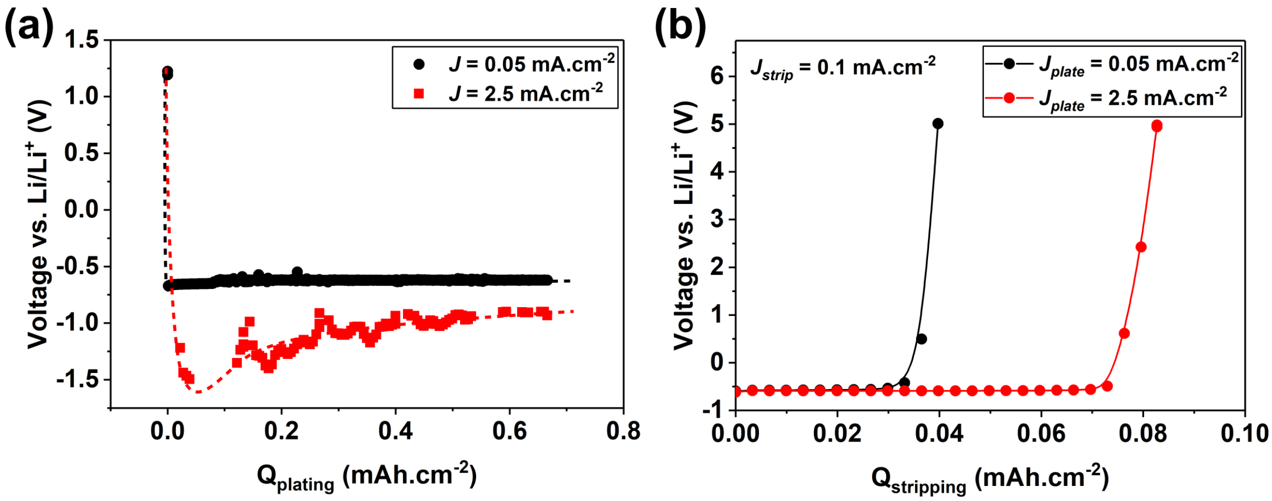

Supplementary Figure 8. **Utilisation efficiency of plated Li anodes.** (a) Potential across the lithium-free cell SS|LPSCl|LiIn measured during passage of ∼0.65 mAh·cm^{-2} of charge, at 2.5 mA·cm^{-2} and 0.05 mA·cm^{-2}. From Figure 4b in the main text, R_{int} was seen to converge to similar values for plating conducted at various current densities after passage of a similar amount of charge. (b) Cell potential measured during stripping of the plated Li in both samples from (a) at a uniform current density of 0.1 mA·cm^{-2}. Only ∼0.04 mAh·cm^{-2} charge could be recovered from sample plated at 0.05 mA·cm^{-2}, as opposed to about twice that amount, ∼0.08 mAh·cm^{-2} from the sample plated at 2.5 mA·cm^{-2}. Since a significant amount of Li is used up for the formation of an SEI layer, the amount of Li recoverable after the initial plating is considerably reduced. However, even the amount of Li recoverable, indicating anode utilisation efficiency, is relatively higher for sample originally plated at a higher current density.

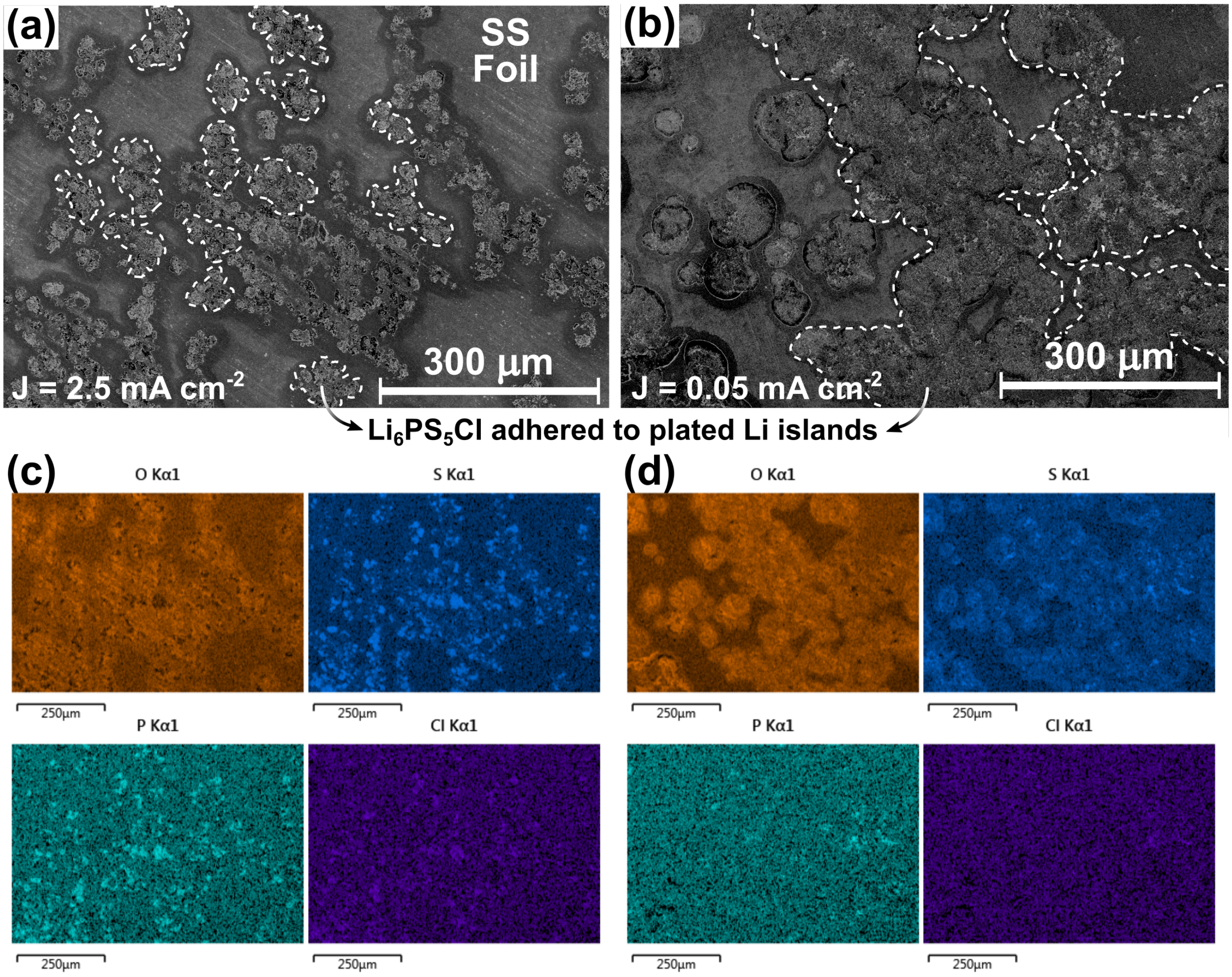

Supplementary Figure 9. **Morphology of plated Li anodes from ex situ SEM.** Ex situ postmortem scanning electron micrographs of stainless steel (SS) foil surface used as a substrate for the Li deposition/stripping at the negative electrode, peeled off from a lithium-free SS|LPSCl|LiIn cell, and corresponding EDX spectral maps for O, S, P and Cl after passing ∼30 μAh·cm^{-2} of charge at (a, c) $J_{2.5}$ = 2.5 mA·cm^{-2}, (b, d) $J_{0.05}$ = 0.05 mA·cm^{-2}. Island-like features seen in (a) and (b) represent LPSCl SE adhered to Li plated at SS foil surface. Contrast in corresponding EDX spectral intensities in (c) and (d) especially for S and P indicate extent of adhesion of LPSCl to underlying plated Li.[3] Higher contrast represents higher adhesion to underlying Li nuclei as in (c). The spatial distribution of Li-SE islands is seen to be more uniform and homogeneous for Li plated at higher current density (c), than at lower current density (d) where Li-SE islands appear coalesced, and sparsely distributed.

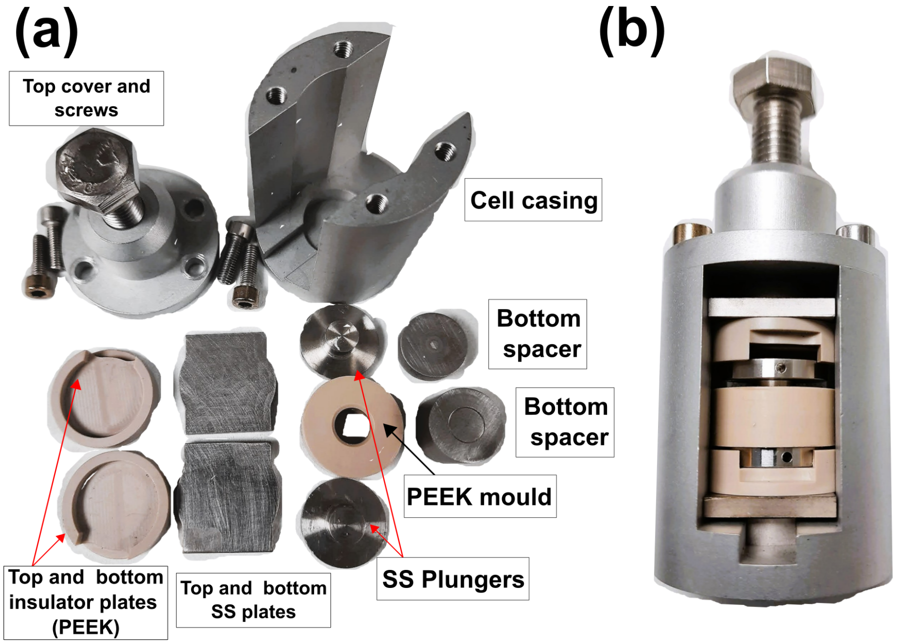

Supplementary Figure 10. Experimental setup for electrochemical testing. (a) Components of cell assembly used for electrochemical characterisation. (b) Photograph of an assembled cell. The pressure on the cell can be adjusted by setting the top-screw using a torque wrench.[4]

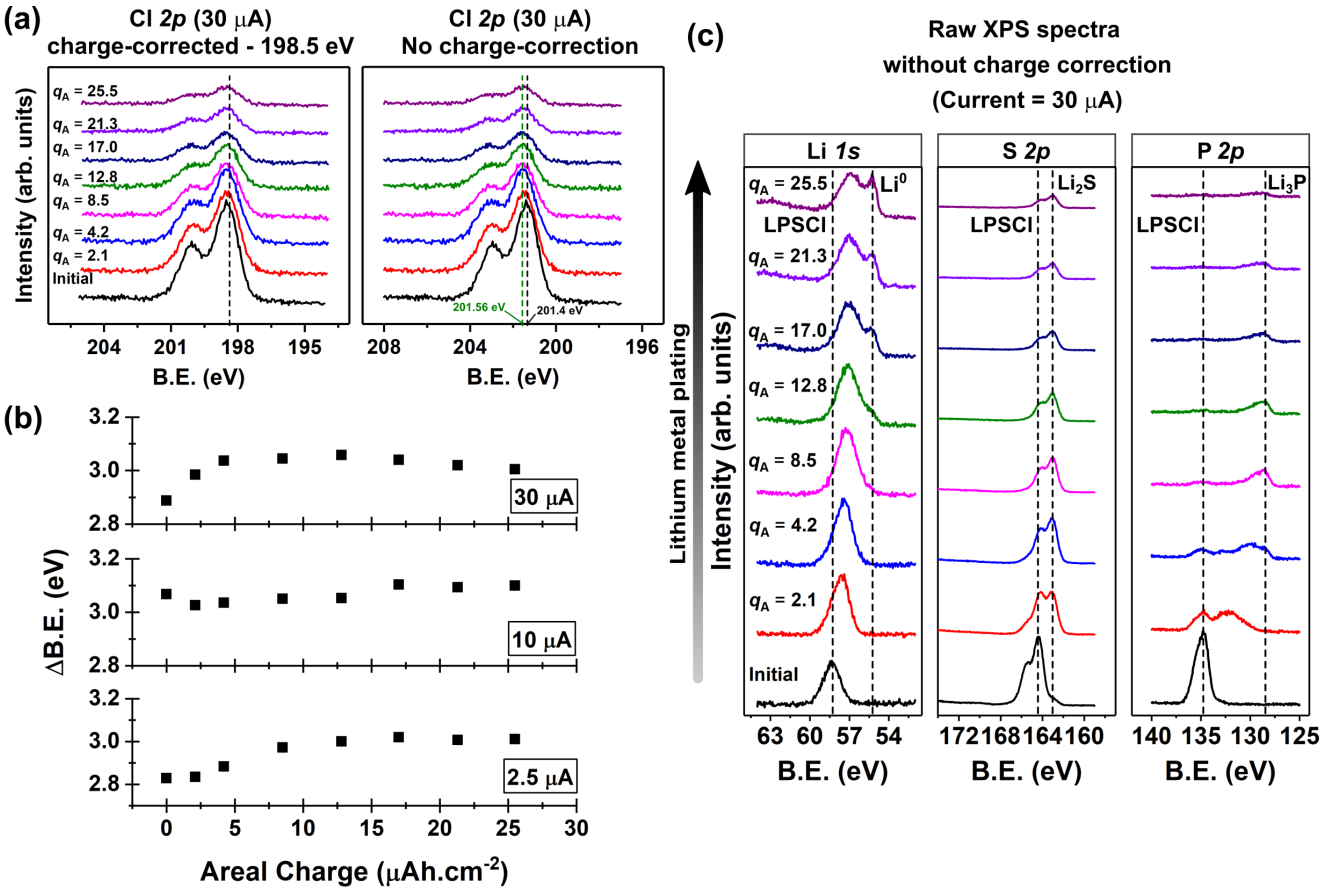

Supplementary Figure 11. XPS spectra charge calibration using Cl *2p* signal. (a) Evolution of XPS spectra pertaining to the Cl *2p* transitions during the virtual electrode plating process at the LPSCl surface, at an applied EBC of 30 μA, with and without charge-correction to the Cl $2p_{3/2}$ peak at 198.5 eV. (b) The computed binding energy shifts after charge correction to Cl $2p_{3/2}$ at 198.5 eV ($B.E_{raw}$-$B.E._{corrected}$) for all three EBCs used for virtual electrode plating (2.5 μA, 10 μA, and 30 μA) as a function of areal charge passed, q_A (μAh·cm^{-2}). (c) Evolution of XPS spectra pertaining to other core level transitions, Li *1s*, S *2p* and P *2p*, for an EBC of 30 μA, as acquired and without post-processing or charge-correction. Error bars in B.E. shifts presented in panel b are computed from associated residual standard deviation between raw data and fitted curves, using CasaXPS.[1]

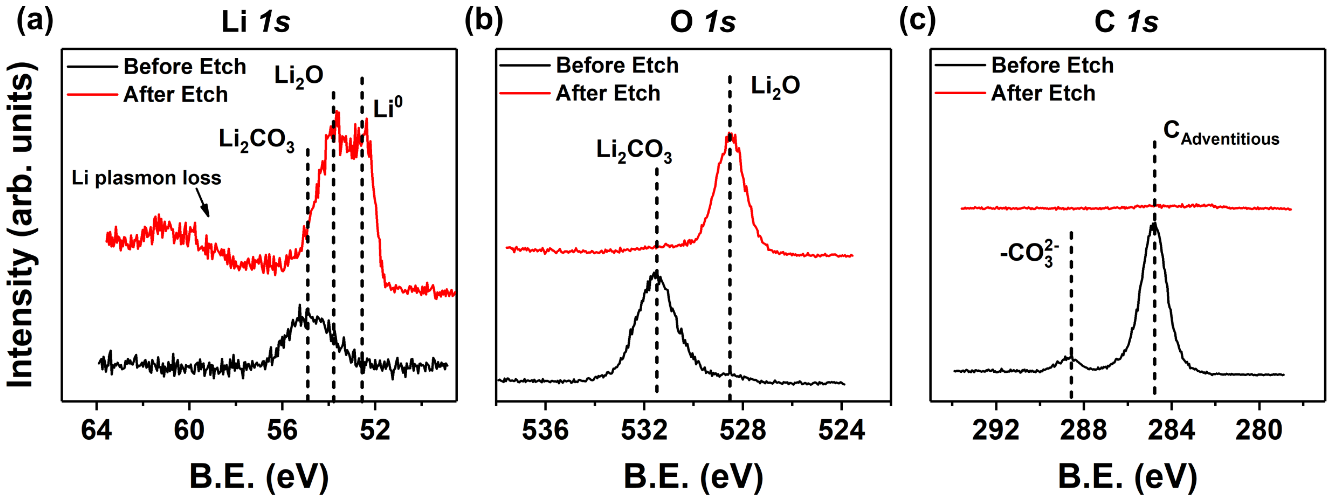

Supplementary Figure 12. XPS of Li foil surface. XPS spectra for scraped and cleaned Li foil used for in situ sputtering in the pristine and etched states (4kV Ar^+ ion beam) for (a) Li *1s*, (b) O *1s*, and (c) C *1s* transitions.

Sl. No.	Solid Electrolyte	Characterisation Method	Technique	Li source	SEI Thickness	Ref.
1	Li_6PS_5Cl	XPS	In situ	In situ sputtering	≈23 nm	2
2	Li_6PS_5Cl	ToF-SIMS + AFM	In situ	Electron-beam induced plating	≈250 ± 25 nm	5
3	Li_6PS_5Cl	XPS	Ex situ	Li foil (Honjo Metal 60 μm) peeled from SE	≈151 nm	3
4	LiPON	Cryo-EM	Ex situ	Evaporation (570 nm) on LiPON-coated substrate	≈80 nm	6

Supplementary Table 1. SEI thickness at Li-SE interfaces. Summary of reported findings on SEI thicknesses in Li-SE interfaces for LPSCl and similar SEs. This indicates a lack of general consensus on the thickness of SEI formed for comparable SE materials.

Current density ($mA \cdot cm^{-2}$)	Charge passed ($\mu Ah \cdot cm^{-2}$)	R_1 (Ω)	Q_2 ($F.s^{a-1}$)	a_2	R_2 (Ω)	Q_3 ($F.s^{a-1}$)	a_3	R_3 (Ω)	Q_4 ($F.s^{a-1}$)	a_4	R_4 (Ω)	s_5 ($\omega \cdot s^{-1/2}$)	Error
2.5	0.00	Not fitted											
	10.68	55.0	8.78E-03	0.41	16.4	6.82E-01	0.77	2.12	1.35E-02	0.56	41.2	1.4	0.32
	21.37	52.3	7.30E-03	0.49	17.0	9.42E-01	0.73	0.83	1.50E-02	0.64	26.8	4.9	0.24
	32.05	50.4	7.55E-03	0.49	16.2	8.66E-04	0.73	0.95	1.44E-02	0.63	25.6	5.1	0.20
	42.74	48.9	8.76E-03	0.49	12.3	9.30E-04	0.73	0.95	1.16E-02	0.60	29.8	5.0	0.16
	53.42	47.7	7.66E-03	0.52	9.3	6.57E-04	0.75	0.96	9.51E-03	0.61	31.8	5.7	0.17
	64.10	46.5	6.46E-03	0.56	6.7	3.66E-04	0.80	0.97	8.04E-03	0.61	35.1	5.7	0.15
	74.79	45.4	7.21E-03	0.54	7.5	6.25E-04	0.76	0.93	7.77E-03	0.63	34.9	5.8	0.14
0.5	0.00	Not fitted											
	10.68	45.6	1.85E-06	0.49	2.1	3.28E-03	1.00	16.78	8.95E-03	0.48	76.1	0.1	0.32
	21.37	43.6	2.40E-06	0.59	2.1	2.28E-03	1.00	7.84	7.06E-03	0.54	53.8	2.8	0.54
	32.05	42.2	3.25E-06	0.61	1.8	2.52E-03	1.00	5.60	6.72E-03	0.57	47.8	3.5	0.65
	42.74	40.8	3.78E-06	0.62	1.7	2.66E-03	1.00	4.95	6.88E-03	0.59	44.8	4.1	0.57
	53.42	39.4	4.42E-06	0.64	1.5	2.73E-03	1.00	4.37	7.09E-03	0.58	43.1	4.1	0.54
	64.10	38.0	5.09E-06	0.65	1.4	2.75E-03	1.00	3.77	7.30E-03	0.58	42.1	3.9	0.54
	74.79	36.8	6.97E-06	0.58	1.1	4.57E-03	0.99	4.10	7.68E-03	0.58	40.6	3.8	0.49
0.05	0.00	Not fitted											
	10.68	56.8	2.99E-05	0.50	16.7	6.14E-03	0.82	33.90	2.01E-03	0.55	47.9	8.7	2.18
	21.37	56.6	1.52E-01	0.45	9.5	1.47E-02	0.84	17.29	2.62E-03	0.56	49.4	6.4	0.79
	32.05	54.2	1.25E-03	0.35	7.6	4.53E-02	1.00	6.98	2.94E-03	0.58	51.3	5.2	1.21
	42.74	51.9	7.42E-03	0.25	8.3	5.76E-02	1.00	6.42	3.13E-03	0.69	45.3	4.4	1.09
	53.42	50.9	1.17E-02	0.26	7.0	7.86E-02	1.00	5.82	3.31E-03	0.60	42.6	3.9	0.93
0.01	0.00	Not fitted											
	16.24	49.9	1.44E-04	1.00	23.3	4.42E-03	0.62	2.49	9.75E-04	0.39	169.7	1.2	1.84
	24.36	49.2	3.45E-04	0.90	25.2	2.04E-03	0.66	3.34	1.37E-03	0.35	139.5	1.3	2.17
	32.48	52.0	3.07E-04	0.74	52.1	4.91E-02	1.00	7.19	2.48E-03	0.31	90.1	1.9	1.74
	40.60	58.9	8.81E-04	0.57	46.9	5.73E-02	1.00	8.53	1.09E-03	0.50	64.7	3.4	1.79
	48.72	59.3	1.49E-03	0.48	55.1	3.90E-02	1.00	7.67	3.84E-04	0.68	44.5	4.9	1.75
	56.84	55.9	8.84E-04	0.46	53.4	5.37E-02	1.00	10.08	8.40E-04	0.72	43.6	2.9	1.62
	64.96	56.3	1.31E-03	0.45	85.5	1.40E-01	0.98	8.16	8.23E-04	0.70	15.1	1.5	1.33

Supplementary Table 2. Table of EIS fitting parameters. List of parameters and their values used to fit EIS measurements taken from SS|SE|LiIn cells at various current densities tested, along with root mean square error computed between raw and fitted data.

Supplementary Notes

Supplementary Note 1:

The binding energies of characteristic transitions of elements probed in this study, such as Cl *2p*, S *2p*, P *2p* and Li *1s*, are <200 eV. This corresponds to kinetic energies >1200 eV for the emitted photoelectrons. This results in relatively long inelastic mean-free paths (λ_{imfp}) for photoelectrons in Li and Li-containing compounds[7], of the order of ~45-55 Å. Further, the effective XPS probing depth ($3\lambda_{imfp}$) at these energies can be ~13-17 nm.[8] Thus, even with a large amount of Li plated, the XPS spectra constitute signals from depths up to 15-20 nm under the surface.

Supplementary Note 2:

In situ sputtering of Li was conducted by varying the Ar^+ ion beam voltage, which controls the Li sputter rate. By comparing the high resolution spectra for Li *1s* (Supplementary Figure 3a), it is evident that the metallic Li (Li^0) feature appears at an estimated film thickness of $t_{Li} \approx 4$ nm, for Li sputtered using Ar^+ beam accelerated to 4 kV, as opposed to when sputtered at 2 kV ($t_{Li} \approx 10$ nm). The fraction of Li^0 component in the Li *1s* spectra estimated from spectral quantification is also evidently higher as shown in Supplementary Figure 3c. Similarly, a comparison of the P *2p* spectra (Supplementary Figure 3b) demonstrates complete reduction of the phosphorous species to Li_3P for $t_{Li} < 4$ nm, when sputtered at 4 kV. This is in contrast to evolution of the same species progressing through partially reduced phases before eventually reducing to P^{3-}, for Li sputtered at 2 kV ($t_{Li} \approx 10$ nm), as seen in Supplementary Figures 3b and 3d. This indicates that reaction kinetics as well as reactant energetics play a critical role in determining SEI formation and evolution.[9]

Supplementary Note 3:

Despite being an UHV (ultra-high vacuum) environment, the XPS chamber ambient contains trace oxygen, moisture and other carbon contaminants. During the virtual electrode plating process, lithium would be expected to gradually react with the existing environment. It is to be noted here that most of the initial contamination present on the surface of the sample can be attributed to the glovebox environment or even the inert transfer vessel used to transfer between the glovebox and the XPS.[10,11] In a separate study, Gibson et al. demonstrate that there are but minor differences in reaction products formed on introduction of Li to the SE surface (by evaporation, or by sputtering, or by a virtual electrode plating setup).[11]

Supplementary Figure 5a shows a comparison of the SE surface composition before and after plating of Li (~25.5 μAh·cm^{-2}). It can be seen that despite slightly varying initial compositions, especially in the case of O and C, the compositions for species present in the SE (Li, S, P and Cl) are either similar or follow a trend that is suggestive of current density-dependent reactivities with Li. Supplementary Figures 5b-d provide more insight into the evolution of composition with increasing plated Li. Even in the case where initial C contamination was relatively large (beam current = 10 μA), trends in composition evolution for Li, S, P and Cl are similar throughout - distinct differences in final amounts of Li emerge along with corresponding differences in S concentration (attributable to Li bound as Li_2S).

Supplementary Note 4:

Supplementary Figure 9 shows an ex situ examination of scanning electron micrographs (SEM) of the SS foil surface peeled off from the LPSCl surface, after passing $q_A \approx 30$ μAh·cm^{-2} of charge at $J_{2.5}$ and $J_{0.05}$. Supplementary Figures 9a and 9b respectively reveal patterns of Li islands covered with SE material akin to those described by Schlenker et al.[3] Herein, the Li nucleation sites can be inferred to be centrally located within the regions marked with dashed curves. These islands also consist of an adjoining region surrounding these spots, followed by a fringe region towards the outer edge of the patterns. As in

Schlenker et al.'s study, the marked regions contain residues of LPSCl adhered to the Li even after peeling, indirectly representing the extent of contact of the SS current collector to the SE surface. For the sample plated at $J_{2.5}$ the islands appear relatively smaller and more uniformly distributed spatially. Meanwhile, energy dispersive X-ray spectral maps of for S, P and Cl Kα_1 transitions (Supplementary Figure 9c) show high apparent contrasts that are representative of enhanced adhesion of SE to the underlying Li nuclei plated on the SS foil. Whereas, for plating conducted at $J_{0.05}$, Supplementary Figure 9b depicts the coalescence of small islands into larger ones showing a more diffuse distribution of SE components in EDX spectral maps (Supplementary Figure 9d). This is indicative of poorer adhesion of SE to underlying Li nuclei, likely resulting from the formation of an SEI that is still evolving with continued passage of charge.

Supplementary Note 5:

Supplementary Table 2 lists out the parameters used to fit EIS measurements taken from SS|SE|LiIn cells at current densities $J_{2.5}$, $J_{2.5}$, $J_{2.5}$ and $J_{0.01}$, depicted in Figure 3a-d of the main text. Based on the equivalent circuit shown in Figure 3a and its interpretation described in Figure 4a of the main text, the EIS curve fit elements R_1, R_2, R_3 and R_4 correspond to R_{Bulk}, R_{GB} (GB = Grain Boundary), R_{SEI} and R_{CT} (CT = charge transfer) respectively, while the components Q_2, Q_3, and Q_4 correspond to the admittance of ideal capacitances contributing to the impedance using the relation $Z_Q = \frac{1}{Q_n(j\omega)^{a_n}}$, for $n = 2,3,4$.[12] Finally, the element s_5 describes the Warburg diffusion admittance, that contributes to the overall impedance through the relation $Z_{W_s} = \frac{1}{s_5\sqrt{j\omega}}$.[12] The table also lists the error estimated using root mean square method (RMSE) between raw and fitted data at specific intervals of charge passed. The data pertaining only to 0 charge passed (initial condition) was not fitted owing to large errors and inconsistencies noticed during fitting, especially since the initial system corresponds to a state that is described by a different equivalent circuit that need not necessarily be comparable with that of the system undergoing Li plating.

Supplementary References

1. Fairley, N. *et al.* Systematic and collaborative approach to problem solving using X-ray photoelectron spectroscopy. *Appl. Surf. Sci. Adv.* **5**, 100112 (2021).
2. Wenzel, S., Sedlmaier, S. J., Dietrich, C., Zeier, W. G. & Janek, J. Interfacial reactivity and interphase growth of argyrodite solid electrolytes at lithium metal electrodes. *Solid State Ionics* **318**, 102–112 (2018).
3. Schlenker, R. *et al.* Understanding the Lifetime of Battery Cells Based on Solid-State Li6PS5Cl Electrolyte Paired with Lithium Metal Electrode. *ACS Appl. Mater. Interfaces* **12**, 20012–20025 (2020).
4. Doerrer, C. *et al.* High Energy Density Single-Crystal NMC/Li 6 PS 5 Cl Cathodes for All-Solid-State Lithium-Metal Batteries. *ACS Appl. Mater. & Interfaces* **13**, 37809–37815 (2021).
5. Otto, S. *et al.* In situ investigation of lithium metal–solid electrolyte anode interfaces with tof-sims. *Adv. Mater. Interfaces* **9**, 2102387 (2022).
6. Cheng, D. *et al.* Unveiling the stable nature of the solid electrolyte interphase between lithium metal and lipon via cryogenic electron microscopy. *Joule* **4**, 2484–2500 (2020).
7. Tanuma, S., Powell, C. J. & Penn, D. R. Calculations of electron inelastic mean free paths. IX. Data for 41 elemental solids over the 50 eV to 30 keV range. *Surf. Interface Analysis* **43**, 689–713 (2011).

8. Greczynski, G. & Hultman, L. X-ray photoelectron spectroscopy: Towards reliable binding energy referencing. *Prog. Mater. Sci.* **107**, 100591 (2020).

9. Tan, D. H. S. *et al.* Elucidating reversible electrochemical redox of li ₆ ps ₅ cl solid electrolyte. *ACS Energy Lett.* **4**, 2418–2427 (2019).

10. Wood, K. N. & Teeter, G. XPS on Li-Battery-Related Compounds: Analysis of Inorganic SEI Phases and a Methodology for Charge Correction. *ACS Appl. Energy Mater.* **1**, 4493–4504 (2018).

11. Gibson, J. *et al.* Gently does it!: In situ preparation of alkali metal - solid electrolyte interfaces for photoelectron spectroscopy. *Faraday Discuss.* (2022).

12. Barsoukov, E. & Macdonald, J. R. *Impedance Spectroscopy: Theory, Experiment, and Applications* (John Wiley and Sons, 2005).
